# Scalable High-Performance Production of Recombinant Horseradish Peroxidase from *E. coli* Inclusion Bodies

**DOI:** 10.3390/ijms21134625

**Published:** 2020-06-29

**Authors:** Diana Humer, Julian Ebner, Oliver Spadiut

**Affiliations:** TU Wien, Institute of Chemical, Environmental and Bioscience Engineering, Research Area Biochemical Engineering, Gumpendorfer Straße 1a, 1060 Vienna, Austria; diana.humer@tuwien.ac.at (D.H.); julian.ebner@tuwien.ac.at (J.E.)

**Keywords:** *E. coli*, inclusion bodies, recombinant horseradish peroxidase, refolding, hydrophobic interaction chromatography, reversed-phase high-performance liquid chromatograpy (RP-HPLC), size exclusion HPLC (SEC-HPLC)

## Abstract

Horseradish peroxidase (HRP), an enzyme omnipresent in biotechnology, is still produced from hairy root cultures, although this procedure is time-consuming and only gives low yields. In addition, the plant-derived enzyme preparation consists of a variable mixture of isoenzymes with high batch-to-batch variation preventing its use in therapeutic applications. In this study, we present a novel and scalable recombinant HRP production process in *Escherichia coli* that yields a highly pure, active and homogeneous single isoenzyme. We successfully developed a multi-step inclusion body process giving a final yield of 960 mg active HRP/L culture medium with a purity of ≥99% determined by size-exclusion high-performance liquid chromatography (SEC-HPLC). The Reinheitszahl, as well as the activity with 2,2′-azino-bis (3-ethylbenzothiazoline-6-sulphonic acid) (ABTS) and 3,3′,5,5′-tetramethylbenzidine (TMB) as reducing substrates, are comparable to commercially available plant HRP. Thus, our preparation of recombinant, unglycosylated HRP from *E. coli* is a viable alternative to the enzyme from plant and highly interesting for therapeutic applications.

## 1. Introduction

Horseradish peroxidase (HRP) (EC 1.11.1.7) has high industrial relevance entailing an immense pool of published data, ranging from refolding of denatured plant enzyme to recombinant production in different hosts [1,2,3,4,5,6,7]. HRP is an oxidoreductase that contains heme as a cofactor, two calcium ions and four disulfide bridges [6,8]. This plant-derived enzyme naturally occurs in the horseradish root (*Armoracia rusticana*), where it is glycosylated usually at eight of nine asparagine sites [9,10]. Due to this glycosylation, plant HRP (pHRP) has a size of 44 kDa, but when the enzyme is produced in *Escherichia coli* (rHRP) it is unglycosylated resulting in a size of only 34.5 kDa [11]. The natural enzyme in *A. rusticana* has a large variety of isoforms, with 34 different entries in the UniProtKB database (April 2020) [1]. To date, HRP for industrial use is still extracted from the plant; therefore, the composition and abundance of the isoenzymes in the final product is diverse. The application range of HRP is immensely versatile; first and foremost, the protein is used as reporter enzyme because it reacts with many substrates to give a chromogenic, fluorogenic or electrochemical signal. Therefore, HRP is used for nucleic acid, antibody and protein labelling in immunoassays, diagnostic kits and microarrays [12,13,14]. The enzyme can also function as a biosensor, which can be applied for the detection of hydrogen peroxide, but also for other substances, such as glucose, L-phenylalanine, ethanol or hydroquinone [15,16,17,18,19,20]. In the field of environmental protection, HRP is used for the bioremediation of pollutants (mostly phenols) and for the decolorization of dyes in the textile industry [21,22,23]. In chemistry, the protein is employed for organic polymer synthesis and biocatalysis [24,25,26,27,28,29]. Another interesting field with high potential is the biomedical use of HRP, for example in medical diagnostics [30,31] or targeted cancer treatment [32,33]. However, these applications require an HRP preparation that complies with pharmaceutical standards, available as a steady supply in large amounts. These requirements are met in a recombinant production strategy, where a single isoenzyme can be produced homogeneously and, if necessary, without glycosylation. As mentioned above, HRP is unglycosylated in *E. coli* and therefore well suited for medical applications, as foreign glycosylation patterns can cause immune reactions in humans [34]. Recombinant protein production in *E. coli* follows two major routes: the protein of interest can either be soluble or insoluble in the form of protein aggregates, also known as inclusion bodies (IBs). In the *E. coli* cytoplasm, HRP forms IBs because the absence of glycans on the protein surface results in enhanced hydrophobicity and the disulfide bridges cannot be formed. Both production strategies pose several advantages as well as disadvantages and are dependent on protein properties as well as cultivation parameters [35,36]. While initially IBs were regarded as undesirable, they show many advantages for production, such as high titers, high purities after recovery and the potential to express proteins that would otherwise be toxic to the host cell [37,38]. However, the downstream process (DSP) is more elaborate as the protein must be refolded to the native state. In 2018, we published a mini-review that summarizes the current developments and analytical tools for refolding [39]. In general, IBs are harvested by centrifugation, followed by a washing step and solubilization with denaturing agents, such as chaotropic substances (urea, guanidine hydrochloride (GuHCl)) or detergents (Triton X-100, sodium dodecyl sulfate). The solubilized protein is then transferred to a buffer where the correctly folded protein is energetically favored over the unfolded protein. This can be done in different ways, with the most common methods being batch dilution, fed-batch dilution and on-column refolding. Depending on the characteristics of the protein of interest, different factors have to be considered for successful refolding. For the correct refolding of peroxidases, the establishment of the redox system and the method, as well as the time point of cofactor addition, are essential. In Table 1 we present a comparison of refolding attempts for different class II and III peroxidases. Concerning HRP, the first refolding experiments were performed more than 30 years ago by Smith et al. [40], where a yield of 3% with 630 U/mg with ABTS as substrate and a protein concentration of 0.057 mg/mL was reported. Grigorenko et al. [3] performed HRP refolding from IBs using a His tagged protein and IMAC (immobilized metal affinity chromatography) as capture step after refolding. The refolding experiment resulted in 6-8 mg HRP/L culture medium with a specific activity of 1160 U/mg for ABTS as reducing substrate.

Asad et al. [41] published a comprehensive investigation on the refolding conditions of horseradish peroxidase, focusing on the buffer system, redox conditions and additives suitable for HRP stabilization. They used one factor at a time and response surface methodology (RSM) to obtain a yield of 3.6 mg HRP from 15 mg solubilized protein. The highest enzyme activity was 10 U/mg with 4-aminoantipyrine as substrate. In 2016, we performed a comparative study on the production of HRP both in a soluble form and from IBs. We obtained a relatively high yield of 150 mg HRP/L culture medium for refolded HRP with a specific activity of 62.5 U/mg (ABTS), but unfortunately, only 15 mg/L could be recovered after concentration with spin filters [44]. The translocation of HRP to the periplasm resulted in a final yield of 28 mg HRP/L culture medium and 12.7 U/mg with ABTS as reducing substrate. Thus, all previous production strategies resulted in low yields and/or decreased enzyme activity and stability. This is also the case for many other peroxidases (Table 1), where the highest yield was reported for Tobacco peroxidase with 79 mg/L culture medium.

In this study we present a novel production process for HRP from *E. coli* IBs with upscaling potential to industrial dimensions. Moreover, we assume that this procedure can be transferred to other peroxidases of the plant peroxidase superfamily, as all enzymes of class II and III are monomers and contain a non-covalently bound heme, two calcium ions and four disulfide bridges. As shown in Table 1, several studies concerning the refolding process of HRP have previously been published, resulting in established ranges for several important parameters, for example the urea concentration during solubilization and refolding. For this study, we focused on the influence of several parameters on the overall process, resulting in an integrated approach. This integrated approach of solubilization and refolding was found to be necessary and highly beneficial. Furthermore, the time of addition and the concentration of hemin had a significant influence on the refolding yield. The best results were obtained with a slow addition of hemin to the apo-enzyme. Previous approaches of HRP purification from IBs used either IMAC, SEC (size-exclusion chromatography) or CEX (cation exchange chromatography) as a capture step after refolding [3,40,41,42,44]. In this study, we developed a novel protocol for salt precipitation and hydrophobic interaction chromatography (HIC). The combination of these optimized conditions resulted in a final process yield of 959 mg active HRP/L culture medium with a purity of ≥99% determined by SEC-HPLC. The enzymatic activity for the substrates ABTS and TMB, as well as the Reinheitszahl (Rz), are comparable to the plant-derived enzyme. The developed process allows the scalable production of the unglycosylated, single isoenzyme HRP C1A at the highest yield reported thus far (Table 1).

## 2. Results and Discussion

In this study, we developed a novel production process for recombinant HRP from *E. coli* inclusion bodies using a series of multivariate experiments and an integrated approach. Table 2 summarizes the parameters and ranges examined for each unit operation.

### 2.1. Solubilization and Refolding

**Figure 1:**
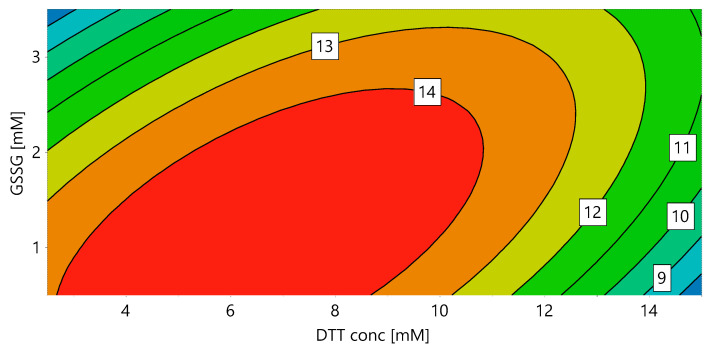
Response contour plot for the volumetric activity (U/mL) with ABTS as substrate, dependent on the DTT concentration in the solubilization mix and the GSSG concentration in the refolding buffer (DoE 1).

In order to identify the optimal redox system for solubilization and refolding, a DoE for DTT and GSSG concentration was performed (“DoE 1”). During solubilization all disulfide bridges should be reduced, granting the complete solubilization of the protein. In the subsequent refolding step, the correct disulfide bridges have to be formed, requiring an oxidizing environment. We hypothesized that these two unit operations are dependent on each other, as the DTT concentration had an influence on the refolding yield even if the solubilization yield stayed constant. In order to provide an integrated approach, the final refolding yield (determined as the volumetric activity) was used as a response for both DTT and GSSG concentration. Figure 1 shows the contour plot for DoE 1, with 7.11 mM DTT and 1.27 mM GSSG resulting in the highest yield for a protein concentration of 0.5 g/L during refolding.

For the used dilution of 1:40 solubilization mix in refolding buffer, this is equivalent to a GSSG:DTT ratio of 7:1 in the refolding mix. The interaction of DTT and GSSG is clearly visible, with increasing DTT requiring increasing GSSG concentrations. This shows the importance of optimizing the redox system in an integrated approach including solubilization and refolding and using the final refolding yield as response. Note that the absolute values of DTT and GSSG presented here might vary depending on the properties of the IBs, which are influenced by several factors, such as fermentation conditions (e.g., temperature, induction time and strength etc.) and unit operations performed before solubilization (e.g., homogenization, separation of IBs from soluble proteins, IB wash and also steps such as freezing and storage).

Based on these results, a second DoE (“DoE 2”) was performed, using DTT, GSSG and protein concentration as factors, and volumetric activity (U/mL) and specific activity (U/mg) as responses. These responses were chosen since lower protein concentrations usually lead to higher refolding yields [62]. However, low protein concentrations require large buffer volumes and accordingly large vessels, which is a drawback for subsequent unit operations. The originating costs are a major downside of industrial IB processes, thus making the protein concentration a highly relevant factor. Initially, we assumed that the optimized redox conditions might be dependent on the protein concentration, with higher protein concentrations requiring higher DTT concentrations during solubilization. Contrary to this hypothesis, the DTT concentration resulting in the highest yield actually decreased for higher protein concentrations, with the GSSG concentration staying almost constant (Table 3). The optimized conditions for 0.5 g/L were 17.2 mM DTT and 2.2 mM GSSG, differing significantly from the results in DoE 1 (7.11 mM DTT and 1.27 mM GSSG). The reason for this is the strong contribution of the protein concentration as a factor, especially for the specific activity (Table 3 and Supplementary Figure S1). It is therefore difficult to predict optimal DTT and GSSG concentrations independent of the protein concentration. While this restricts the use of this particular experimental design for the optimization of the redox system, it clearly shows the importance of the investigation of the protein concentration during refolding. The highest specific activity in DoE 2 was achieved at a concentration of 0.5 g/L. All higher protein concentrations resulted in lower refolding yields (lower specific activities (U/mg)), therefore requiring a larger amount of IBs for the same amount of correctly folded HRP. The volumetric activity showed the opposite trend, rising with the concentration of protein in the refolding mix, with the highest volumetric activity achieved at 9.4 mM DTT, 2.2 mM GSSG and 1.6 g/L protein (26.9 U/mL) (Supplementary Figure S2). As expected, the specific activity at these conditions was low, with only 16.8 U/mg, which might result in problems during the subsequent capture step due to high concentrations of incorrectly folded protein. Furthermore, these conditions required three times more IBs for only a 20% increase in refolding yield. Based on these results, a protein concentration of 0.5 g/L during refolding was chosen for subsequent experiments. However, for an industrial production process the economically most feasible protein concentration might be different. For known USP (upstream process) and DSP costs, the presented models can be used to calculate the sweet spot between low refolding yields (requiring more IBs and raising the cost of the USP) and low concentrations of correctly folded HRP after refolding (requiring larger buffer volumes during refolding and subsequent capture steps resulting in higher DSP costs).

Asad et al. [41] previously reported that “the effect of the pH on HRP refolding was not significant over the range of 7–10”. These experiments were performed with a one factor at a time approach and the concentrations of DTT and GSSG were kept constant for all tested pH values. However, the reaction kinetics of DTT and GSSG are pH dependent, with slow reaction kinetics for pH values < 9. In order to cover this pH dependency of the redox pair, we performed a CCF DoE with the factors DTT concentration, GSSG concentration and pH value (pH 7–10) with the volumetric activity as response (“DoE 3”). The protein concentration during refolding was kept constant at 0.5 g/L for all experiments. Refolding at pH 7 resulted in very low refolding yields for all combinations of DTT and GSSG, so that the pH value was the dominant factor for the model. This, in turn, led to bad predictions for DTT and GSSG at pH 8.5 and 10. Therefore, the experiments at pH 7 were excluded from the model in order to obtain a correct depiction of the influence of DTT and GSSG concentrations at higher pH values. Comparing pH 8.5 and pH 10, different DTT and GSSG concentrations were required in order to achieve maximum refolding yields, underlining the importance of the multivariate approach (Supplementary Figure S3). The overall highest refolding yield was achieved at 6.7 mM DTT and 1.26 mM GSSG and pH 10, which resulted in an increase of 25% compared to refolding at pH 8.5. Furthermore, at pH 10, deviations of the DTT and/or GSSG concentration have less influence on the refolding yield. Based on the results of DoE 2 and DoE 3, we chose 7.11 mM DTT, 1.27 mM GSSG, 0.5 g/L protein concentration in the refolding buffer and pH 10 as the optimized conditions for solubilization and refolding.

The experiments in DoEs 1–3 were all performed in 2 mL reaction tubes, resulting in a maximum volumetric activity of 56 U/mL after refolding. In order to identify possible scale-up effects, these optimized conditions were scaled up to a refolding vessel with a volume of 1200 mL (“refolding vessel experiment 1”), resulting in a higher volumetric activity of 84 U/mL. Due to the tendency of protein aggregation during refolding, we assumed that favorable stirring conditions and surface to volume ratio led to this increase in activity for the upscaled process. Overall, the presented optimization approach resulted in a scalable and robust process with high refolding yields.

### 2.2. Hemin Addition

The concentration and time of hemin addition is another important parameter during refolding. Hemin has to be supplied in order to obtain holo-HRP; however, it is not necessary for the correct formation of apo-HRP. Previous studies showed that higher refolding yields could be achieved if hemin was added after the refolding step [41,42,44,63]. We suspect that this is the case due to the hydrophobic nature of hemin, promoting aggregation if added early on during refolding. Therefore, we assumed the time point of hemin addition, as well as the concentration, to have an influence on the refolding yield. The first experiments to test this hypothesis were performed using 2 mL reaction tubes, and optimized conditions were then validated in a bench-scale refolding vessel.

**Figure 2:**
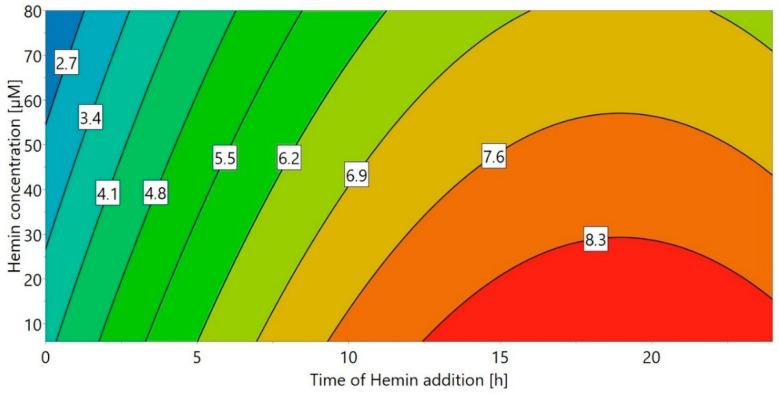
Response contour plot for DoE 4 with different times of hemin addition and hemin concentration as factors and the volumetric activity (U/mL) as response.

A DoE approach (“DoE 4”) was chosen for the small-scale experiments, using the concentration of hemin as well as the time of hemin addition as factors. The volumetric activity (U/mL) after refolding was used as the response (Figure 2). When hemin was added immediately after refolding start (0 h), the refolding yield strongly depended on the hemin concentration, with higher hemin concentrations leading to an over 10-fold reduced yield. This effect was decreased for later hemin addition times—in fact, when hemin was added 24 h after the start of the refolding process, the influence of the hemin concentration was negligible. This is most likely a result of the hydrophobic nature of hemin, promoting aggregation of unfolded HRP, especially early during the refolding process. Therefore, the optimal time for hemin addition appears to be 6 h or longer after the start of refolding, with the maximum refolding yield achieved for a concentration of 6 µM hemin after around 19 h. Although 6 µM hemin was sufficient and higher hemin concentrations did not lead to an increase of the refolding yield, 20 µM were chosen for all further experiments in order to prevent hemin to become a limiting factor for potential higher refolding yields in the bench-scale refolding vessel.

**Figure 3:**
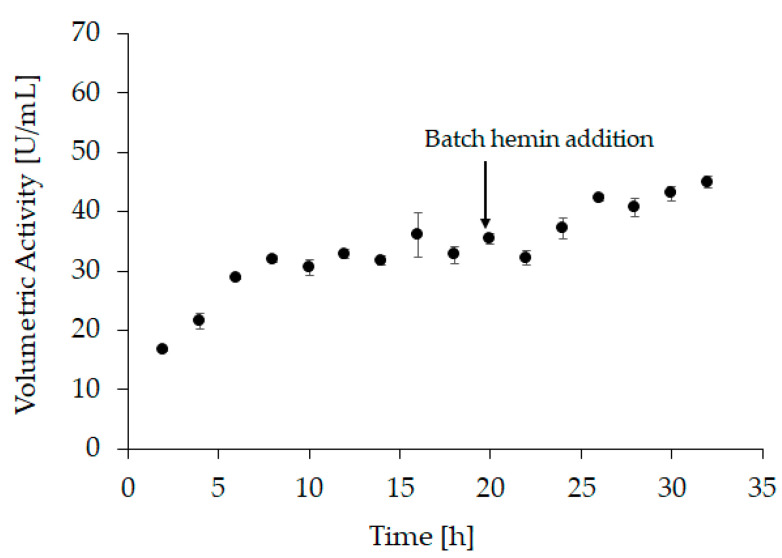
At-line sampling of “refolding vessel experiment 2” with volumetric activity in U/mL as response, samples 1–9 (2 h to 18 h) were taken before hemin was added to the refolding vessel therefore, 20 µM were added afterwards and samples were incubated for another 2 h before activity measurement. Samples 10–16 (20 h to 32 h) were drawn after hemin addition but were still incubated for 2 h before activity measurement. All samples were measured in triplicates, with an average standard deviation < 6%.

The optimal conditions were transferred to a bench-scale refolding vessel with a refolding volume of 1200 mL (“refolding vessel experiment 2”). For this experiment, hemin was added after 20 h of refolding to a final concentration of 20 µM. Additionally, the refolding kinetics was monitored during this experiment. Samples were taken every 2 h to measure the activity at-line (Figure 3 and Supplementary Table S1). However, since hemin is required to form holo-HRP, 20 µM hemin were added to each sample immediately after sampling and the activity was measured after incubation for two hours. After the addition of hemin to the refolding vessel (i.e., after 20 h), samples were still taken every 2 h, but no further hemin was added. Still, the samples were incubated for 2 h before measurement. As we see in the at-line activity measurement, refolding was completed after approximately 8 h. These observations are in good agreement with the results obtained from small-scale experiments and underline the importance of addition of hemin after the refolding process is finished.

**Figure 4:**
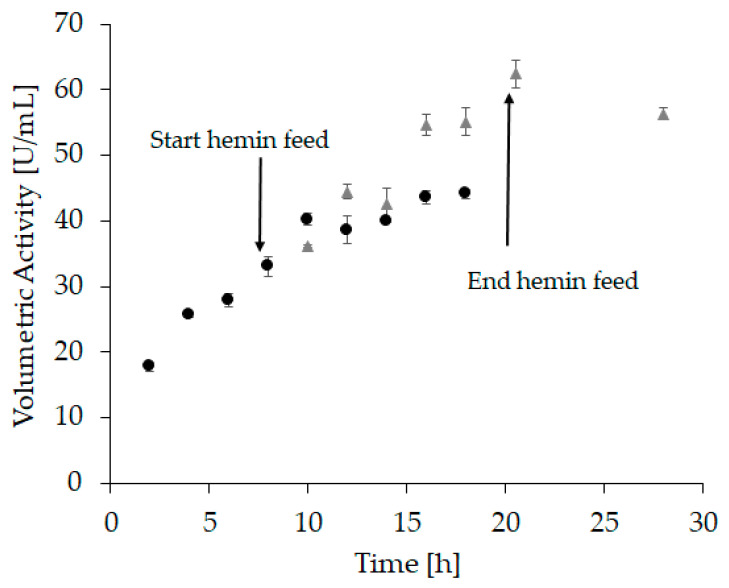
At-line sampling of “refolding vessel experiment 3” with volumetric activity (U/mL) as response. The hemin feed was started after 8 h and lasted 12 h. Circles represent samples taken before and after the start of the feed, where hemin was added to each sample to reach a final concentration of 20 µM. Samples were incubated for another 2 h before activity measurement. Triangles represent samples that were measured directly after the samples were drawn with no further hemin addition. All samples were measured in triplicates, with an average standard deviation < 6%.

Based on these experiments, we assumed that a linear hemin feed starting after complete apo-enzyme formation might further improve the refolding yield. The hypothesis was that hemin is prone to self-aggregation as well as aggregation with incorrectly folded protein, leading to a reduced amount of hemin available to form holo-HRP. Moreover, hemin aggregates could potentially impede further downstream applications, e.g., through irreversible binding to chromatography columns. A steady supply of small amounts on the other hand can immediately be used for holo-enzyme formation while aggregates are kept at a minimum. Therefore, in “refolding vessel experiment 3” a linear feed commencing 8 h after refolding starts with a total feeding time of 12 h was applied. Again, samples were taken every two hours (Figure 4 and Supplementary Table S2).

At the beginning of the hemin feed, the volumetric activity (Figure 4, sample 4) was similar to the activity measured in “refolding vessel experiment 2” (Figure 3, sample 4). This demonstrates a good reproducibility and comparability between these two experiments. After an incubation time of 12 h with hemin, the volumetric activity in refolding vessel experiment 2 reached 45 U/mL (Figure 3, sample 16; Supplementary Table S1), whereas it was significantly higher after 12 h of hemin feed with 62 U/mL (Figure 4, sample 6 grey triangles; Supplementary Table S2). Moreover, the refolding time was shortened, as the maximum volumetric activity for “refolding vessel experiment 2” was reached after 44 h (46.5 U/mL, Supplementary Table S1), whereas this was achieved already after 20.5 h in this experiment (62 U/mL, Supplementary Table S2). These data substantiated our hypothesis that a linear hemin feed leads to an increased refolding yield.

All results presented above were done at pH 8.5, since we performed these experiments after optimizing the redox system, but before, we identified pH 10 as most beneficial during refolding (Supplementary Figure S3). Therefore, we wanted to verify the applicability of these results at pH 10. Applying a hemin feed instead of batch addition at pH 10 in fact resulted in a 23% increase of the refolding yield. We therefore concluded that the effect of our hemin addition strategy was independent of the pH value.

### 2.3. Capture and Concentration

The last unit operation investigated in this study was the capture step after refolding. Several different approaches, including IMAC, CEX and SEC, have been reported thus far [3,40,41,42,44]. In this study, a novel approach using HIC was developed. HIC is suitable for other peroxidases [46,47], but has never been used for HRP purification before. This approach turned out to be highly beneficial because the binding conditions require high salt concentrations, which precipitated impurities, such as excess hemin and protein aggregates, while correctly folded HRP was stable in solution at high salt concentrations. This has the advantage that impurities are already separated before the capture step, resulting in a higher binding capacity as well as easier cleaning and regeneration of the chromatographic resin.

First, we compared (NH_4_)_2_SO_4_ and NaCl for protein precipitation with the goal of reaching efficient impurity separation and high HRP recovery in the supernatant at the same time. Due to their position in the Hofmeister series, the concentration was varied between 0 M and 1.5 M for (NH_4_)_2_SO_4_ and 0 M and 4 M for NaCl, respectively. The recovery of the volumetric activity after protein precipitation was 96% for 1 M (NH_4_)_2_SO_4_ and 95% for 4 M NaCl. Specific activity increased 2.5-fold for (NH_4_)_2_SO_4_ and 4.5-fold for NaCl (Table 4). Due to the higher purification factor and the compatibility for potential medical applications in the future, we chose NaCl as salt.

After salt precipitation, we tested different HIC resins, pH values and elution strategies (Supplementary Figures S4–S8) and finally used a resin with medium hydrophobicity (HiTrap Butyl FF), a pH of 8.5 and a step elution strategy. Using this strategy, active HRP was concentrated more than 9-fold resulting in a final concentration of 0.5 g/L active HRP in the eluate (Table 5) with a purity ≥ 99%.

### 2.4. pH 8.5 vs. pH 10

Although we clearly demonstrated that refolding at pH 10 leads to the highest refolding yield, two final process runs at pH 8.5 and pH 10 were performed in order to compare the overall process yields. As demonstrated in small-scale and several bench-scale experiments, the optimal redox potential for HRP refolding was established with a GSSG:DTT ratio of 7:1. The final refolding time was 19 h, with 8 h refolding before the start of a 10 h hemin feed (final concentration 20 µM). When refolding was done at pH 10, the refolding mix was brought to pH 8.5 before 4 M NaCl were added for salt precipitation. After centrifugation, the HRP solution was captured on a HiTrap Butyl FF column and eluted at 75% buffer B (Supplementary Figures S7 and S8).

Table 6 summarizes the results for refolding at pH 8.5 compared to pH 10. Refolding at pH 10 significantly increased the refolding yield to 74% when compared to 44% at pH 8.5. In addition, the total yield of HRP was enhanced 1.7-fold and reached 959 mg/L *E. coli* cultivation broth. The produced recombinant HRP had a very high purity (≥99%) and the specific catalytic activity with ABTS as substrate was around 1500 U/mg for both pH 8.5 and pH 10.

### 2.5. Characterization of Refolded HRP

Finally, the kinetic parameters of the commercially available plant HRP from Sigma-Aldrich (pHRP; Cat. No.: P6782) were compared to refolded HRP (rHRP; Table 7). In fact, both enzyme preparations were comparable in terms of catalytic activity underlining the applicability and high potential of the recombinant HRP from *E. coli* inclusion bodies.

### 2.6. Final HRP Production Process

The final process steps to prepare recombinant HRP from *E. coli* inclusion bodies are summarized in Table 8.

## 3. Materials and Methods

### 3.1. Chemicals

GSSG was purchased from AppliChem (Darmstadt, Germany). ABTS was purchased from AppliChem or Sigma-Aldrich (St. Louis, MO, USA). Plant HRP Type VI-A (Cat. No.: P6782), hemin (hemin from bovine, ≥90%) and TMB were purchased from Sigma-Aldrich. DTT and all other chemicals were purchased from Carl Roth (Karlsruhe, Germany).

### 3.2. Strain and Growth Conditions

The *hrp* gene coding for HRP variant C1A was codon-optimized for *E. coli* and obtained from GenScript USA Inc. (Piscataway, NJ, USA). The plasmid pET21d+ was used for HRP IB production in the cytoplasm. A stop codon was introduced so that the protein was produced without any tags. HRP was produced in *E. coli* BL21(DE3) in a 10 L Biostat Cplus stainless steel bioreactor (Sartorius, Germany). The pre-culture was grown in 0.5 L DeLisa medium [64] at 37 °C, 230 rpm in a 2.5 L Ultra Yield™ Flask (UYF; Thomson Instrument company, Encinitas, CA, USA) over night. Subsequently, the pre-culture was added to 4.5 L DeLisa medium in the bioreactor vessel and batch fermentation at 35 °C was run for 6 h. The pH was adjusted constantly to 7.2 and the dissolved oxygen was kept above 20%. During the 16 h fed-batch phase the specific uptake rate (*q_s_*) was 0.333 g/g/h, which was set to 0.25 g/g/h after induction with 0.5 mM isopropyl-β-D-thiogalactopyranoside (IPTG). After an induction phase of 8 h, the biomass was harvested by centrifugation and stored at −20 °C until further processing. All data collection and control of the process was done using a process information management system (Lucullus; Biospectra; Schlieren, Switzerland). All IBs used in this study were produced in one fermentation run.

### 3.3. Homogenization and Wash

The biomass was resuspended using an IKA T10 basic ULTRA-TURRAX (Staufen, Germany) in 5 mL buffer A/g wet biomass (Buffer A: 50 mM Tris/HCl; pH 8; 500 mM NaCl; 1.5 mM ethylenediaminetetraacetic acid (EDTA)) and homogenized at >1200 bar, three passages, cooled, using a GEA Niro Soavi Panda PLUS (Düsseldorf, Germany). The homogenized suspension was centrifuged (15,650 g; 20 min, 4 °C), the supernatant discarded and the cell debris resuspended in 10 mL buffer B/g wet cell debris (Buffer B: 50 mM Tris/HCl; pH 8; 500 mM NaCl; 2 M Urea) and centrifuged again (15,650 g; 20 min, 4 °C). This washing step with buffer B was performed twice. Afterwards, the pellet was resuspended in water (5 mL water/g wet cell debris), the suspension aliquoted into pre-weighed 50 mL reaction tubes, centrifuged (15,650 g; 20 min, 4 °C) and the pellets were stored at −20 °C.

### 3.4. Solubilization

For solubilization, an aliquot of the washed and frozen IBs was thawed, the wet inclusion body (wIB) weight was determined and the pellet was resuspended in the appropriate solubilization buffer to reach a wIB concentration of 100 g/L (solubilization buffer 1: 50 mM Tris/HCl; pH 8.5; 6 M Urea; solubilization buffer 2: 50 mM Glycine; pH 10; 6 M Urea). After resuspension, DTT was added to reach a final concentration in the solubilization mix of 1 mM–28.44 mM and the solubilization mix was incubated (room temperature (RT); 0.5 h; slight agitation), followed by centrifugation (20,379 g; 20 min; 4 °C). The supernatant was immediately used for refolding, and the pellet was discarded.

### 3.5. Refolding

#### 3.5.1. Small-Scale

Small-scale experiments were performed using DoE approaches. Planning and analysis of DoEs were done using Umetrics MODDE 10 (Malmö, Sweden). All small-scale refolding experiments were performed using 2 mL reaction tubes. The solubilisate was diluted 1:40 in the appropriate refolding buffer (refolding buffer 1: 20 mM Tris/HCl; pH 8.5; 2 M Urea; 2 mM CaCl_2_; 7% *v*/*v* glycerol; varying GSSG concentrations; refolding buffer 2: 20 mM glycine; pH 10; 2 M urea; 2 mM CaCl_2_; 7% *v*/*v* glycerol; varying GSSG concentrations), followed by incubation at 4 °C; 48 h; with slight agitation. A 1 mM hemin stock solution was prepared in 100 mM potassium hydroxide (KOH).

#### 3.5.2. DoE 1: Redox Conditions

For the first DoE, the DTT concentration for solubilization (solubilization buffer 1; pH 8) and the GSSG concentration in the refolding buffer (refolding buffer 1; pH 8.5) were varied (Table 9 and Supplementary Table S4). A CCF with the volumetric activity after refolding as a response was used and four replicates were performed for the center point (8.75 mM DTT and 2 mM GSSG).

#### 3.5.3. DoE 2: Protein Concentration during Refolding

For the second DoE, the DTT concentration for solubilization (solubilization buffer 1; pH 8), the GSSG concentration in the refolding buffer (refolding buffer 1; pH 8.5) and the protein concentration in the refolding mix were varied (Table 10 and Supplementary Table S5). A CCF with the volumetric and specific activity after refolding as a response was used and four replicates were performed for the center point (14.22 mM DTT, 2.54 mM GSSG and 1 g/L protein concentration).

#### 3.5.4. DoE 3: Redox Conditions and pH

For the third DoE, the DTT concentration for solubilization, the GSSG concentration in the refolding buffer and the pH of the solubilization and refolding buffer were varied (Table 11 and Supplementary Table S6). For pH 8.5, solubilization and refolding buffers 1 were used; for pH 10, solubilization and refolding buffers 2 were used. A CCF with the volumetric activity after refolding as a response was used and four replicates were performed for the two center points (7.11 mM DTT and 2.54 mM GSSG for both pH 8.5 and pH 10).

#### 3.5.5. DoE 4: Hemin Addition

For this DoE, time and concentration of hemin addition were varied between 0–24 h after refolding start and 6–80 µM hemin, respectively (Table 12). Solubilization buffer 1 (pH 8) and refolding buffer 1 (pH 8.5) were used for all experiments. The volumetric activity was used as response.

### 3.6. Refolding Vessel

#### 3.6.1. Refolding Vessel Set-Up

For bench-scale refolding an Infors Labfors 5 (Bottmingen, Germany) with a vessel volume of 3.6 L was used. All data collection and control of the process was done using Lucullus (Biospectra, Schlieren, Switzerland). Temperature was kept constant at 10 °C during refolding using a Lauda Alpha R8 thermostat (Lauda, Königshofen, Germany) connected to the double jacket of the glass vessel. The temperature was monitored using a sensor connected to the Infors Labfors 5. In addition, pH, dO2 (dissolved oxygen) and redox potential were monitored. The redox potential was monitored using a Hamilton EasyFerm Plus ORP Arc 425 (Hamilton, Bonaduz, Switzerland). The hemin feed was applied using a LAMBDA PRECIFLOW peristaltic liquid pump (LAMBDA laboratory instruments, Switzerland) in combination with a Sartorius Entris scale (Sartorius, Germany), enabling a PID-Feed forward control using Lucullus. Final refolding volumes for the vessel were kept constant at 1200 mL (30 mL solubilizate and a dilution of 1:40).

#### 3.6.2. Refolding Vessel Experiment 1

For this experiment, the solubilization mix contained 7.11 mM DTT and the refolding buffer contained 1.27 mM GSSG. Solubilization buffer 2 (pH 10) and refolding buffer 2 (pH 10) were used. Hemin was added 20 h after refolding start to a final concentration of 20 µM and then incubated for another 5 h, resulting in a total refolding time of 25 h.

#### 3.6.3. Refolding Vessel Experiment 2

For this experiment, the solubilization mix contained 7.11 mM DTT and the refolding buffer contained 1.27 mM GSSG. Solubilization buffer 1 (pH 8) and refolding buffer 1 (pH 8.5) were used. Hemin was added 20 h after refolding start to a final concentration of 20 µM. Samples were taken every 2 h, hemin was added to a final concentration of 20 µM (only for samples taken before hemin addition; the samples taken after hemin addition already contained 20 µM), the samples were incubated (2 h; 4 °C, slight agitation), and then enzyme activity was measured.

#### 3.6.4. Refolding Vessel Experiment 3

For this experiment, the solubilization mix contained 7.11 mM DTT and the refolding buffer contained 1.27 mM GSSG. Solubilization buffer 1 (pH 8) and refolding buffer 1 (pH 8.5) were used. A constant feed (2 mL of a 1 mM hemin stock/h; final concentration 20 µM hemin) was applied 8 h after refolding start until 20 h (12 h feed time). Samples were drawn every 2 h and activity was measured. After the start of the hemin feed, samples were measured both directly (with a low hemin concentration at the start of the hemin feed), and again after the addition of hemin to a final concentration of 20 µM hemin and incubation for 2 h.

### 3.7. Capture and Concentration

HIC was used as a capture step after refolding and protein precipitation. An ÄKTA Pure system (GE Healthcare, Chicago, IL, USA) was used. Three wavelengths (214 nm, 280 nm and 404 nm) as well as the conductivity were monitored. Two different salts were tested for the preceding precipitation step, namely (NH_4_)_2_SO_4_ and NaCl. Several concentrations of both salts were tested in 2 mL reaction tubes (data not shown). The results obtained for the small-scale experiments were then validated using bench-scale experiments with a volume of 150 mL. The salt was slowly added under continuous stirring within 10 min, the solution was then incubated while stirring for 20 min at RT and then centrifuged (20,379 g; 20 min; 22 °C). The capture step was developed in five HIC experiments using different resins, pH values and elution profiles (HIC experiments 1–3 are described in the Supplementary data).

#### 3.7.1. HIC Experiment 4 (Small-Scale)

The load was prepared by adding 267 g NaCl/L refolding mix. A 1 mL HiTrap Butyl FF column (GE Healthcare) was used with a flow rate of 75 cm^−1^·h^−1^. The column was equilibrated with buffer A (20 mM Bis-Tris pH 7; 7% *v*/*v* glycerol; 4 M NaCl) and 49 mL load were applied. After the load, a washing step with 8 column volumes (CV) buffer A was performed. Thereafter, a step elution was performed with 25% buffer B (20 mM Bis-Tris pH 7; 7% *v*/*v* glycerol/ 8 CV), 75% buffer B (10 CV) and 100% (17 CV) buffer B. Volumetric enzyme activity (U/mL) and protein concentration were measured for all fractions. The purity of the active pool was determined using SEC-HPLC and the Reinheitszahl.

#### 3.7.2. HIC Experiment 5 (Scale-Up)

After refolding at pH 10, the pH was adjusted to 8.5 with 2 M HCl under stirring. Afterwards, 267 g NaCl/L refolding mix were added. A column packed with 80 mL Butyl Sepharose 4 Fast Flow (GE Healthcare) was used. The column was equilibrated at a flow rate of 90 cm^−1^·h^−1^ with buffer A (20 mM Bis-Tris pH 7; 4 M NaCl) and 751 mL load were applied. After the load, a wash step with 20% buffer B (20 mM Bis-Tris pH 7/1.5 CV) was performed. Thereafter, a step elution was performed at 75 cm^−1^·h^−1^ with 75% buffer B (3 CV) and 100% (3 CV) buffer B. Volumetric enzyme activity (U/mL) and protein concentration were measured for all fractions. The purity of the active pool was determined using Rz.

#### 3.7.3. Analytics

Enzyme activity: HRP enzyme activity was measured with a Tecan Infinite M200 PRO (Männedorf, Switzerland) using flat-bottom polystyrene 96-well plates. Depending on the concentration of correctly folded HRP, samples were diluted 1:50–1:200 in dilution buffer (20 mM Bis-Tris pH 7; 7% *v*/*v* glycerol). 170 µL of ABTS solution (5 mM ABTS in 50 mM KH_2_PO_4_ pH 5) were mixed with 10 µL of diluted sample in the well, after which 20 µL of hydrogen peroxide (1 mM final concentration) were added to start the reaction. Immediately afterwards, the change of absorption at 420 nm over 2 minutes was recorded (at 30 °C). The volumetric enzyme activity was calculated using the following Equation (1):

(1) $$A\;\left[U/mL\right]=\frac{V_{total}*\Delta A/min*dilution}{V_{sample}*d*\varepsilon }$$

*V_total_* … total volume in cuvette in (μL)
∆*A/min* … change in absorption (∆*A*bs 420 nm/min)
*Dilution* … dilution of the sample
*V_sample_* … volume of sample (µL)
*d* … length of the beam path through the cuvette (*d* = 0.58 cm)
*ε* … extinction coefficient (*ε*_420_ = 36 mM^−1^ cm^−1^ [65]).

Determination of kinetic parameters for final HRP preparation: The procedure was performed as described in [66]. Enzyme activity parameters were determined for the substrates ABTS and TMB in a 96-well plate assay using a Tecan Infinite M200 PRO instrument. For the measurements with ABTS, the reaction mixture in each well contained a saturating hydrogen peroxide concentration of 1 mM and 7 mM ABTS in 50 mM phosphate-citrate buffer, pH 5, in a final volume of 200 µL. The protein sample (5 µL) was mixed with 175 µL ABTS-buffer mixture and the reaction was started with 20 µL of a 10 mM hydrogen peroxide solution. The increase in absorption was followed at 420 nm at 30 °C for 120 s. For the determination of the kinetic parameters, the ABTS concentration was varied (0.1–7 mM) and calculations were performed with the Sigma Plot software (Systat Software INC., San Jose, CA, USA) and an extinction coefficient of *ε*_420_ = 36 mM^−1^·cm^−1^ [65]. For the measurements with TMB, the reaction mixture contained a saturating hydrogen peroxide concentration of 1 mM and varying TMB concentrations (0.02–0.55 mM) in 50 mM phosphate-citrate buffer, pH 5, with a final volume of 200 µL. An extinction coefficient of *ε*_652_ = 39 mM^−1^·cm^−1^ was used [67].

Protein concentration: Protein concentration was determined using the method according to Bradford [68]. In total, 200 µL Bradford solution were mixed with 5 µL sample and the change in absorbance at 595 nm was measured with a Tecan Infinite M200 PRO instrument over the course of 10 min.

RP-HPLC: The HRP concentration in the samples was measured with RP-HPLC using a Polyphenyl BioResolve-RP-mAb 2.7 μm 3.0 × 100 mm column (Waters, MA, USA). The method was run for 10 min with the following program: 25% line B for 0.5 min, 55% line B in a linear gradient for 8 min, 55% line B for 0.5 min and then 25% line B for 1 min (Line A:MilliQ water with 0.1% trifluoroacetic acid (TFA); line B: acetonitrile with 0.1% TFA) at a flow rate of 1.2 mL/min. The column was kept at a constant temperature of 75 °C and the wavelengths 214 nm, 280 nm and 404 nm were monitored.

SEC-HPLC: Purity of HRP was measured using a SEC-HPLC with a BEH 200A SEC 1.7 µm 4.6 × 300 mm, 3.5 µm (Waters, MA, USA) column. The method was run at 0.3 mL/min using 100% line A (Line A: 80 mM phosphate buffer pH 6.8; 250 mM KCl) for 18 minutes. The column was kept at a constant temperature of 30 °C and the wavelengths 214 nm, 280 nm and 404 nm were monitored.

Reinheitszahl (Rz): The Reinheitszahl was calculated as the ratio of absorbance at 404 nm to 280 nm and the absorbance measurement was performed using a Hitachi Double Beam Spectrophotometer U-2900 (Tokyo, Japan).

## Figures and Tables

**Table 1:** Overview of the yield and specific activity of class II and III plant peroxidases refolded from inclusion bodies.

Enzyme	Yield (% or mg/L Culture)	Specific Activity (U/mg)	Reference
HRP	3%	630 U/mg (ABTS) 14500 U/mg (pyrogallol)	Smith et al. [40]
HRP	6-8 mg/L	1160 U/mg (ABTS)	Grigorenko et al. [3]
HRP	24%	10 U/mg (4-aminoantipyrine)	Asad et al. [41]
HRP	16.7 mg/L	4000 U/mg (ABTS)	Gazaryan et al. [42]
HRP	20 mg/L	2000 U/mg (ABTS)	Gazaryan et al. [43]
HRP	15 mg/L	62.5 U/mg (ABTS)	Gundinger et al. [44]
CWPO_C ^1^	0.2 mg/L	1000 U/mg (ABTS)	Kim et al. [45]
CWPO_C ^1^	27.3%	1066 U/mg (syringaldazine) 120 U/mg (guaiacol)	Shigeto et al. [46]
rAtPrx71	28%	1291 U/mg (syringaldazine)	Shigeto et al. [47]
rAtPrx25	30.3%	270 U/mg (guaiacol)	Shigeto et al. [47]
TOP ^2^	79 mg/L	2950 U/mg (ABTS)	Zakharova et al. [48]
LDP ^3^	16.8 mg/L	70.7 U/mg (TMB) 580.7 U/mg (H_2_O_2_)	Fattahian et al. [49]
TOP ^2^	4.6 mg/L	1100 U/mg (ABTS)	Hushpulian et al. [50]
ATP N ^4^	13 mg/L	n.m.	Teilum et al. [51]
BP1 ^5^	9.4 mg/L	n.m.	Teilum et al. [51]
LiP H8 ^6^	1%	39 µmol of veratryl alcohol ox/min/mg of protein	Doyle et al. [52]
VPL2 ^7^	5.5 mg/L	n.m.	Pérez-Boada et al. [53]
LiP H2 ^6^	3.4 mg/L	n.m.	Nie et al. [54]
VBPO ^8^	40 mg/L	550 U/mg (bromination of monochlorodimedone)	Coupe et al. [55]
LiP ^6^	0.38 mg/L	16,300 U/mg (ABTS)	Miki et al. [56]
DyP ^9^	1.5 mg/L	247 U/mg (ABTS)	Linde et al. [57]
MnP ^10^	2.4%	12.9 U/mg (oxidation of Mn^2+^ to Mn^3+^)	Wang et al. [58]
LiP H2 ^6^	2.4%	55.6 U/mg (veratryl alcohol)	Lee et al. [59]
MnP ^10^	0.275 mg/L	140 U/mg (oxidation of Mn^2+^ to Mn^3+^)	Whitwam et al. [60]
BnPA ^11^	29 mg/L	981 U/mg (ABTS)	Rodríguez-Cabrera et al. [61]

^1^ Cationic cell wall peroxidase from *Populus alba L;* ^2^ Tobacco peroxidase; ^3^ *Lepidium draba* peroxidase; ^4^ *Arabidopsis thaliana* peroxidase N; ^5^ Barley grain peroxidase; ^6^ Lignin peroxidase; ^7^ *Pleurotus eryngii* versatile peroxidase; ^8^ Vanadium-dependent bromoperoxidase; ^9^ Dye-decolorizing peroxidase; ^10^ Manganese peroxidase; ^11^ Turnip acidic peroxidase; n.m., not mentioned.

**Table 2:** Overview of investigated unit operations and the corresponding process parameters.

Unit Operation	Parameters	Range
Solubilization	DTT	2.5 mM–28.44 mM
	Protein concentration	20 g/L–80 g/L
	pH	7–10
Refolding	GSSG	0.4 mM–3.5 mM
	Protein concentration	0.5 g/L–2 g/L
	pH	7–10
	Time of hemin addition	0 h–24 h after refolding start
	Hemin concentration	6 µM–80 µM
Salt precipitation	Type of salt	NaCl, (NH_4_)_2_SO_4_
	Salt concentration	0 M–4 M
Capture step HIC	Hydrophobicity of resin	Octyl, Butyl, Phenyl
	pH value (load)	8.5, 10
	Type of elution	Step gradient, linear gradient

DTT, dithiothreitol; GSSG, gluthatione disulfide; NaCl, sodium chloride; (NH_4_)_2_SO_4_, ammonium sulfate.

**Table 3:** Optimized DTT and GSSG concentrations for different protein concentrations based on the model obtained in DoE 2. The factor contribution is shown in brackets.

Protein Conc. (g/L)	Optimized DTT (mM)	Optimized GSSG (mM)	Specific Activity (U/mg)	Volumetric Activity (U/mL)
0.5 (93.2%)	17.19 (1.5%)	2.16 (5.3%)	45.1	22.5
1 (91.5%)	13.49 (1.8%)	2.17 (6.7%)	26.6	26.6
1.5 (88.5%)	9.83 (2.5%)	2.18 (9.0%)	18.9	28.4
2 (81.0%)	7.11 (6.0%)	2.21 (13.0%)	13.7	27.3

**Table 4:** Volumetric and specific activity as well as protein concentration and purification factor for salt precipitation using either 1 M (NH_4_)_2_SO_4_ or 4 M NaCl.

Salt	Volumetric Activity (U/mL)	Protein Conc. (g/L)	Specific Activity (U/mg)	Purification Factor
(NH_4_)_2_SO_4_ 1 M	38.0	0.12	317	2.5
NaCl 4 M	44.3	0.09	492	4.5

**Table 5:** Volume, protein concentration, specific activity and purification factor for salt precipitation followed by hydrophobic interaction chromatography.

	Volume (mL)	Protein Conc. (mg/mL)	Specific Activity (U/mg)	Purification Factor
Refolding end	n.a.	0.51	126	1
Load (after salt precipitation)	50	0.09	726	5.8
Active HRP fraction	4	0.50	1176	9.4

n.a., not applicable.

**Table 6:** Comparison of refolding at pH 8.5 and pH 10.

Process Variables	pH 8.5	pH 10
Specific activity (U/mg)	1507 ± 13	1468 ± 24
Purity SEC-HPLC (%)	≥99	≥99
Refolding yield (%)	44	74
Pure HRP/L culture medium (mg)	562	959
Rz	3.7	4.3
Total Units/refolding vessel (7 mM ABTS)	146700	209500
Overall yield active HRP per 100 mg expressed protein (mg)	19	28

The refolding yield was determined by the final amount of HRP in the refolding mix as a percentage of the total amount of IBs that was solubilized.

**Table 7:** Comparison of the commercially available plant HRP with refolded HRP concerning the kinetic parameters for the substrates ABTS and TMB.

		**ABTS**		
	Vmax (U/mg)	Km (mM)	kcat (s^−1^)	kcat/Km (mM^−1^·s^−1^)
pHRP	1285 ± 70	0.70 ± 0.14	734 ± 41	1043 ± 215
rHRP	1411 ± 43	0.49 ± 0.06	823 ± 25	1677 ± 205
		**TMB**		
	Vmax (U/mg)	Km (mM)	kcat (s^−1^)	kcat/Km (mM^−1^·s^−1^)
pHRP	7446 ± 528	0.101 ± 0.020	4343 ± 308	42830 ± 8864
rHRP	7146 ± 355	0.105 ± 0.014	4169 ± 207	39582 ± 5661

**Table 8:** Unit operations and the respective process parameters for the final production process of HRP from *E. coli* inclusion bodies.

Unit operation.	Parameters	Final Conditions
Solubilization	DTT	7.11 mM
	Protein concentration	20 g/L
	pH	10
Refolding	GSSG	1.27 mM
	Protein concentration	0.5 g/L
	pH	10
	Time of hemin addition	8 h after refolding start
	Hemin concentration	20 µM
Salt precipitation	pH	Adjust to pH 8.5 with 2 M HCl
	Type of salt	NaCl
	Salt concentration	4 M (pH 8.5)
Capture step HIC	Hydrophobicity of resin	Butyl
	pH value (load)	8.5
	Type of elution	Step gradient

**Table 9:** DTT and GSSG concentrations used to optimize the redox conditions in a DoE CCF approach with the volumetric activity as response.

DTT conc. (mM in Solubilizate)	GSSG (mM in Refolding Buffer)
2.5	0.5
8.75	2
15	3.5

**Table 10:** DTT, GSSG and total protein concentrations were used to investigate interactions between the redox system and the protein concentration in the refolding mix.

DTT (mM in Solubilizate)	GSSG (mM in Refolding Buffer)	Protein in the Refolding Mix (g/L)
7.11	1.27	0.5
14.22	2.54	1
28.44	5.08	2

**Table 11:** DoE 3 investigated the interaction between the redox system and the pH during solubilization and refolding using a CCF approach.

DTT (mM in Solubilizate)	GSSG (mM in Refolding Buffer)	pH
2.5	0.4	7
7.11	1.27	8.5
11.72	3.01	10

DTT and GSSG concentrations as well as the pH value of the solubilization and refolding buffers were used as factors.

**Table 12:** Time of addition and concentration of hemin in the refolding mix were used as factors in DoE 4.

Hemin Addition (Time after Refolding Start) (h)	Final Hemin Concentration (µM)
0	6
6	20
12	40
24	80

## Supplementary Materials

The following are available online at https://www.mdpi.com/1422-0067/21/13/4625/s1.

## Abbreviations

ABTS	2,2′-azino-bis (3-ethylbenzothiazoline-6-sulphonic acid)
CaCl_2_	Calcium chloride
CCF	Central composite face centered
CEX	Cation exchange chromatography
CV	Column volumes
dO2	Dissolved oxygen
DoE	Design of experiments
DSP	Downstream process
DTT	Dithiothreitol
GSSG	Glutathione disulfide
GuHCl	Guanidine hydrochloride
HIC	Hydrophobic interaction chromatography
HPLC	High-performance liquid chromatography
IB	Inclusion body
IMAC	Immobilized metal affinity chromatography
pHRP	Plant horseradish peroxidase
rHRP	Recombinant Horseradish Peroxidase
RP	Reversed-phase
RT	Room temperature
Rz	Reinheitszahl
SEC	Size exclusion chromatography
TFA	Trifluoroacetic acid
TMB	3,3′,5,5′-tetramethylbenzidine
USP	Upstream process
wIB	Wet inclusion body
