# Supplementary material for: Scalable High-Performance Production of Recombinant Horseradish Peroxidase from E. coli Inclusion Bodies
Source: Int J Mol Sci. 2020 Jun 29;21(13):4625. doi: 10.3390/ijms21134625 (PMC7369975; doi:10.3390/ijms21134625)
Supplement: Supplementary file 1 [file ijms-21-04625-s001.pdf]

Supplementary data

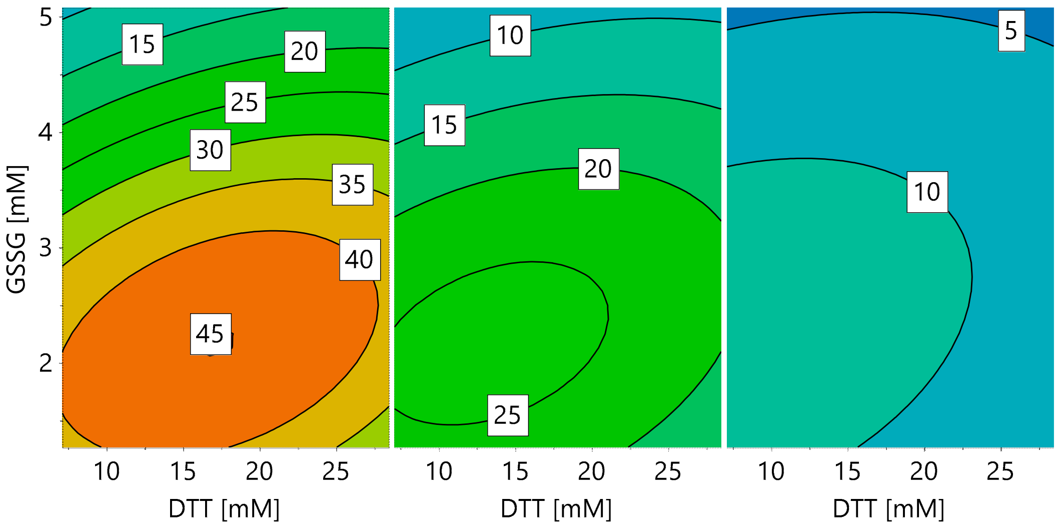

Supplementary Figure S1. Contour plots for 0.5 g/L (left), 1 g/L (middle) and 2 g/L (right) protein concentration in the refolding mix in DoE 2. The response used for this model was the specific activity, which is representative of the relative refolding yield.

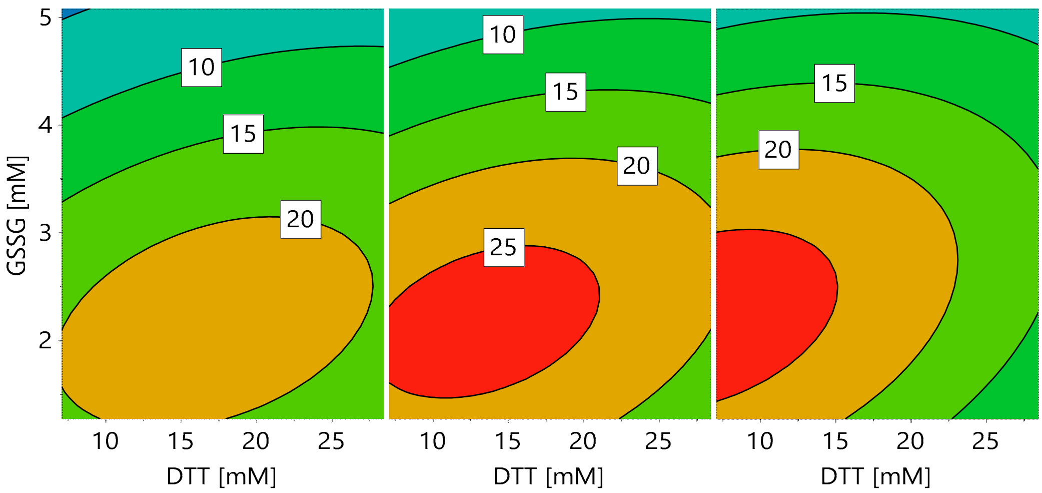

Supplementary Figure S2. Contour plots for 0.5 g/L (left), 1 g/L (middle) and 2 g/L (right) protein concentration in the refolding mix in DoE 2. The response used for this model was the volumetric activity, which is representative for the absolute refolding yield.

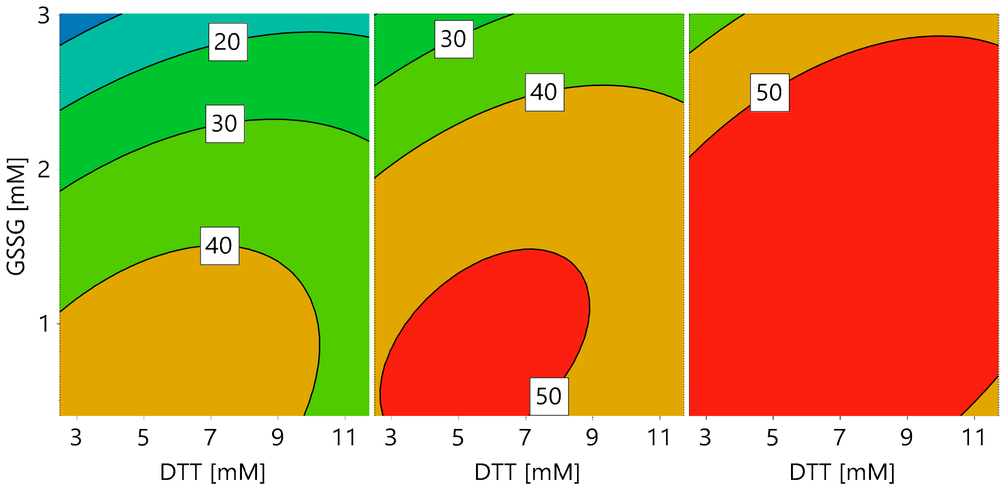

Supplementary Figure S3. Response contour plots for pH 8.5 (left), pH 9.25 (middle) and pH 10 (right) for different DTT and GSSG concentrations (both in mM) in DoE 3.

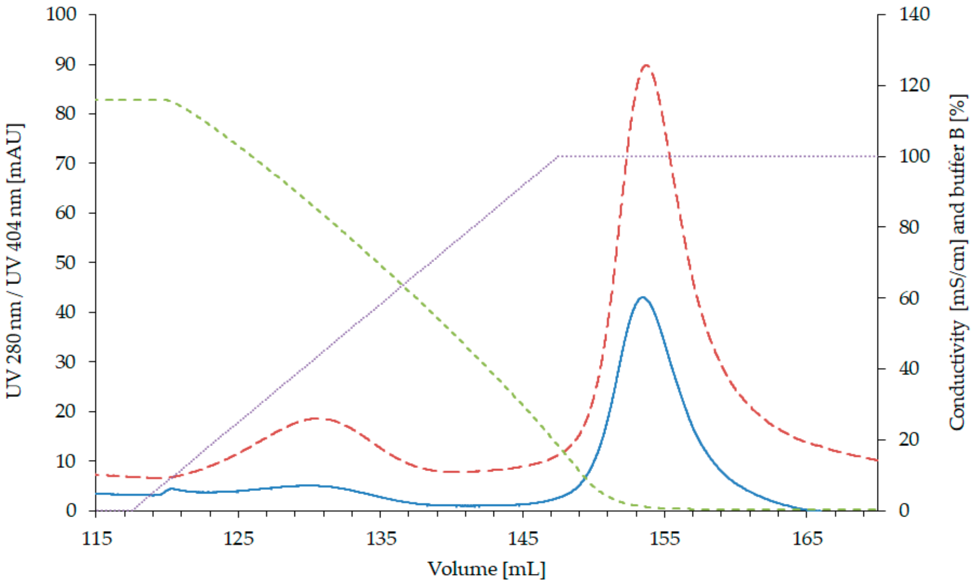

Supplementary Figure S4. HI chromatography profile using a HiTrap Octyl FF column. HRP, refolded at pH 8.5, elutes during the linerar gradient with buffer B and hydrophobic impurities elute at 100 % buffer B. Solid line, UV 280 nm [mAU]; long dashed line, UV 404 nm [mAU]; dotted line, buffer B [%]; short dashed line, conductivity [mS/cm].

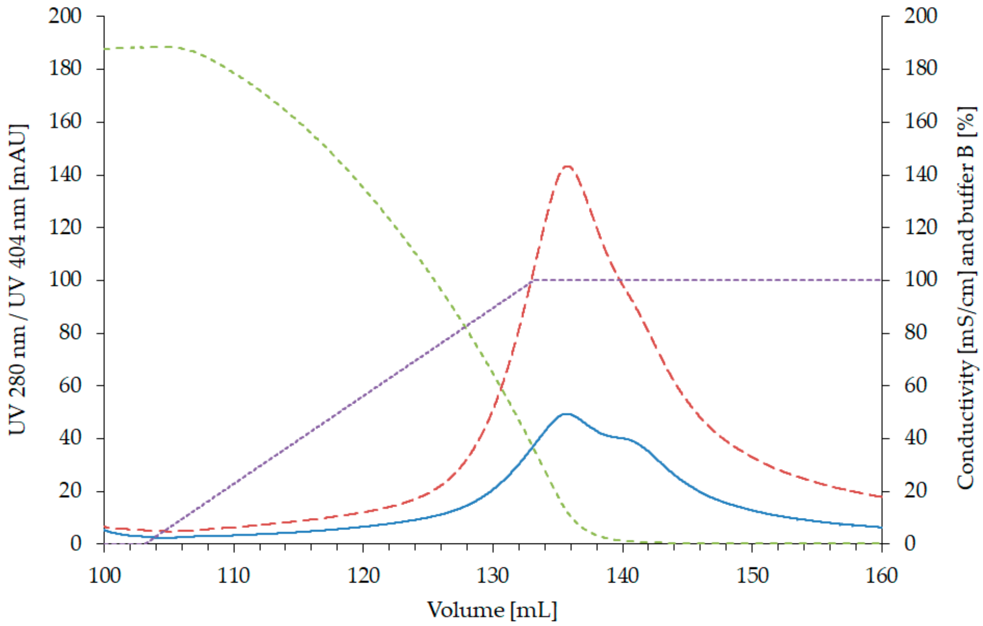

Supplementary Figure S5. Elution of active HRP and impurities using a linear gradient with a HiTrap Phenyl FF column. Active HRP elutes late in the gradient (around 90 % buffer B), leading to an overlap with the elution of hydrophobic impurities, which elute at 100 % buffer B. Solid line, UV 280 nm [mAU]; long dashed line, UV 404 nm [mAU]; dotted line, buffer B [%]; short dashed line, conductivity [mS/cm].

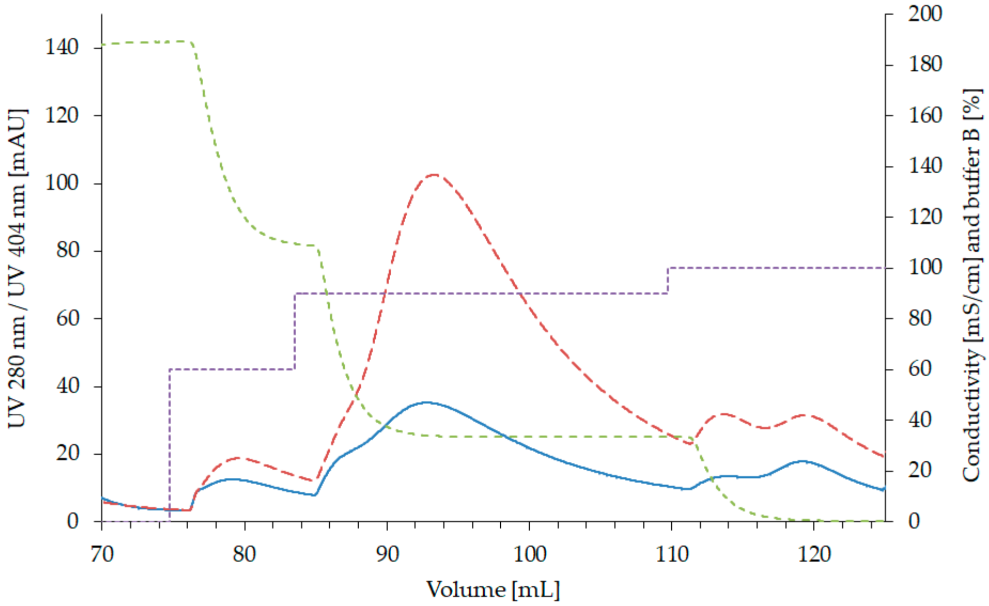

Supplementary Figure S6. Elution of active HRP and impurities using a linear gradient with a HiTrap Phenyl FF column. While separation of active HRP from hydrophobic impurities is possible using 90 % buffer B, this leads to a strong tailing of the active HRP peak. Solid line, UV 280 nm [mAU]; long dashed line, UV 404 nm [mAU]; dotted line, buffer B [%]; short dashed line, conductivity [mS/cm].

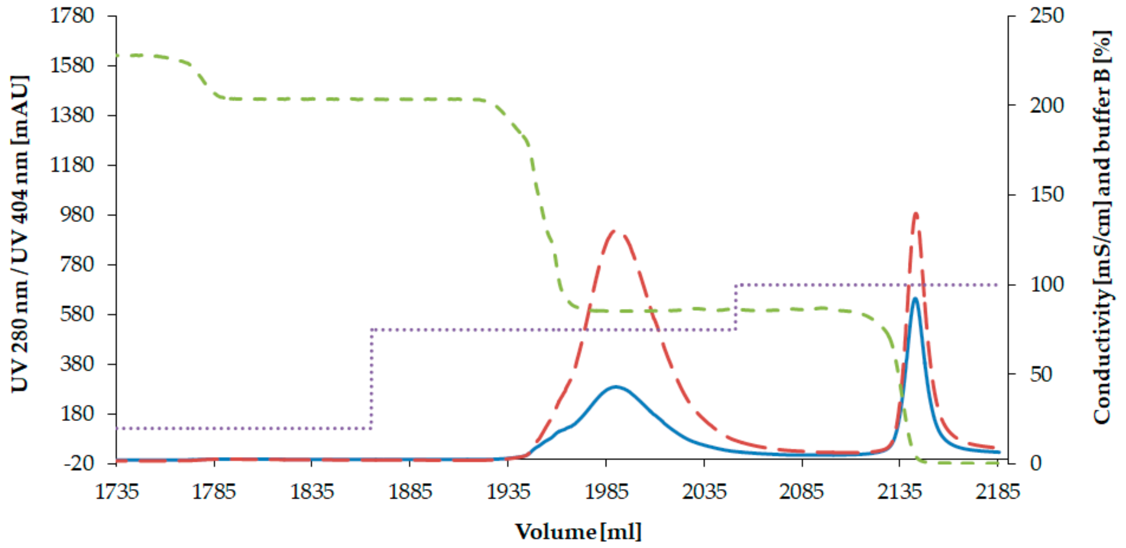

Supplementary Figure S7. Elution of active HRP and impurities using a step gradient with a HiTrap Butyl FF column. Refolding was performed at pH 8.5. The enzyme elutes at 75 % buffer B and hydrophobic impurities elute at 100 % buffer B. Solid line, UV 280 nm [mAU]; long dashed line, UV 404 nm [mAU]; dotted line, buffer B [%]; short dashed line, conductivity [mS/cm].

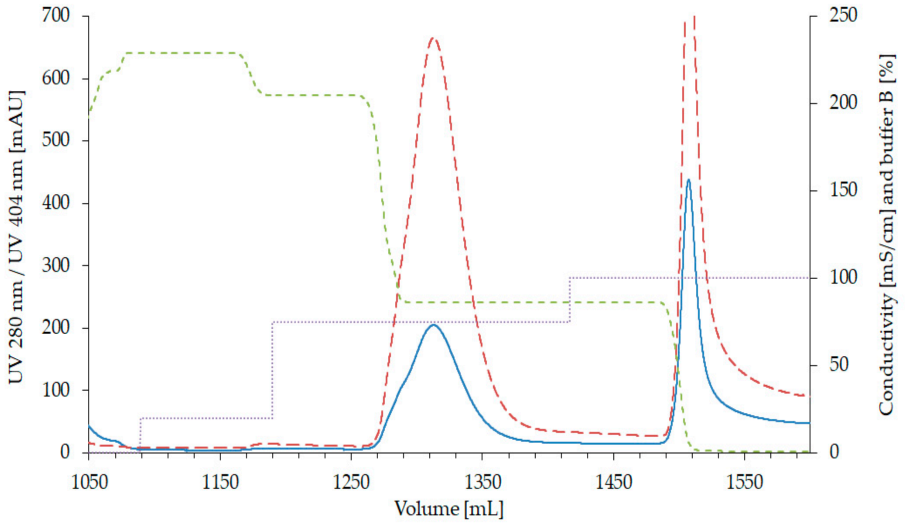

Supplementary Figure S8. Elution of active HRP and impurities using a step gradient with a HiTrap Butyl FF column. Refolding was performed at pH 10. Then pH was adjusted to pH 8.5 before salt precipitation. Active HRP elutes at 75 % buffer B, while hydrophobic impurities elute at 100 % buffer B. Solid line, UV 280 nm [mAU]; long dashed line, UV 404 nm [mAU]; dotted line, buffer B [%]; short dashed line, conductivity [mS/cm].

Supplementary Table S1. Sampling scheme of "refolding vessel experiment 2" with volumetric activity (vAct) inU/mL as a response. Samples 1-9 were taken before hemin was added to the refolding vessel, therefore 20 μM were added afterwards and samples were incubated for another 2 h before activity measurement. Samples 10–17 were drawn after hemin addition but were still incubated for 2 h before activity measurement.

Sample No.	Time from Refolding Start [h]	Volumetric Activity [U/mL]
**Addition of hemin to a final concentration of 20 μM and 2 h incubation before measurement**		
1	2	16.63 ± 0.08
2	4	21.54 ± 1.36
3	6	28.87 ± 0.24
4	8	31.97 ± 0.52
5	10	30.63 ± 1.35
6	12	32.86 ± 0.87
7	14	31.74 ± 0.81
8	16	36.02 ± 3.70
9	18	32.67 ± 1.51
**Hemin already in the refolding vessel and 2 h incubation before measurement**		
10	20	35.44 ± 0.86
11	22	32.18 ± 1.26
12	24	37.24 ± 1.78
13	26	42.31 ± 0.60
14	28	40.73 ± 1.52
15	30	43.05 ± 1.16
16	32	44.96 ± 0.93
17	44	46.45 ± 0.12

Supplementary Table S2. Sampling scheme of "refolding vessel experiment 3" with volumetric activity [U/mL] as a response. The hemin feed was started after 8 h and lasted 12 h. Samples a1-a9 were taken before and after the start of the feed and hemin was added to reach a final concentration of 20 μM. Samples were incubated for another 2 h before activity measurement. Samples b1–b8 were measured immediately, therefore the hemin concentration in the first samples was lower, b6–b8 were measured after the end of the feed containing 20 μM hemin.

Sample No.	Time from Refolding Start [h]	Volumetric Activity [U/mL]
**Addition of hemin to a final concentration of 20 μM and 2 h incubation before measurement**		
a1	2	17.81 ± 0.67
a2	4	25.64 ± 0.40
a3	6	27.86 ± 1.04
a4	8	33.10 ± 1.49
a5	10	40.26 ± 0.93

a6	12	38.64 ± 2.10
a7	14	39.97 ± 0.21
a8	16	43.50 ± 1.01
a9	18	44.10 ± 0.67
	**Samples measured immediately without further hemin addition**	
b1	10	36.11 ± 0.17
b2	12	44.48 ± 1.02
b3	14	42.65 ± 2.43
b4	16	54.71 ± 1.61
b5	18	55.13 ± 2.15
b6	20.5	62.40 ± 2.10
b7	28	56.34 ± 0.93
b8	47.5	50.62 ± 1.93
b9	52	50.56 0.23

Supplementary Table S4. Factor combinations of the DTT concentration in the solubilization mix and GSSG concentration in the refolding mix used for the experimental design of DoE 1. For the center point (8.75 mM DTT and 2 mM GSSG) four replicates were performed.

DTT [mM]	GSSG [mM]	Volumetric Activity [U/mL]
2.5	0.5	13.6
2.5	2	12.3
2.5	3.5	7.3
8.75	0.5	14.5
8.75	2	14.4
8.75	2	13.9
8.75	2	14.0
8.75	2	15.7
8.75	3.5	12.3
15	0.5	7.1
15	2	10.7
15	3.5	10.9

Supplementary Table S5. Factor combinations of the DTT concentration in the solubilization mix, the GSSG concentration in the refolding mix and the protein concentration during refolding used for the experimental design of DoE 2. For the center point (14.22 mM DTT, 2.54 mM GSSG and 1 g/L protein concentration) four replicates were performed.

DTT [mM]	GSSG [mM]	Protein conc. [g/L]	Volumetric Activity [U/mL]	Specific Activity [U/mg]
7.11	1.27	0.5	19.0	38.1
7.11	1.27	1	26.3	26.3
7.11	1.27	2	25.3	12.7
7.11	2.54	0.5	18.4	36.8

7.11	2.54	1	22.4	22.4
7.11	2.54	2	24.3	12.1
7.11	5.08	0.5	5.2	10.4
7.11	5.08	1	6.1	6.1
7.11	5.08	2	8.6	4.3
14.22	1.27	0.5	19.3	38.6
14.22	1.27	1	22.9	22.9
14.22	1.27	2	21.5	10.8
14.22	2.54	0.5	19.0	38.0
14.22	2.54	1	23.4	23.4
14.22	2.54	1	27.8	27.8
14.22	2.54	1	29.2	29.2
14.22	2.54	1	29.9	29.9
14.22	2.54	2	25.9	12.9
14.22	5.08	0.5	6.7	13.4
14.22	5.08	1	7.0	7.0
14.22	5.08	2	10.1	5.1
28.44	1.27	0.5	17.3	34.6
28.44	1.27	1	15.9	15.9
28.44	1.27	2	9.1	4.6
28.44	2.54	0.5	18.7	37.4
28.44	2.54	1	21.4	21.4
28.44	2.54	2	18.1	9.0
28.44	5.08	0.5	7.6	15.3
28.44	5.08	1	9.3	9.3
28.44	5.08	2	8.2	4.1

Supplementary Table S6. Factor combinations of the DTT concentration in the solubilization mix, the GSSG concentration in the refolding mix and the pH value during solubilization and refolding used for the experimental design of DoE 3. For the center points (7.11 mM DTT and 2.54 mM GSSG) four replicates were performed at both pH 8.5 and pH 10.

DTT [mM]	GSSG [mM]	pH	Volumetric Activity [U/mL]
2.5	0.4	10	59.4
2.5	0.4	8.5	42.1
2.5	1.27	8.5	30.8
2.5	1.27	10	61.8
2.5	3.01	8.5	9.6
2.5	3.01	10	29.6
7.11	0.4	8.5	45.5
7.11	0.4	10	57.4
7.11	1.27	8.5	40.5
7.11	1.27	8.5	43.3
7.11	1.27	8.5	45.9
7.11	1.27	8.5	46.0
7.11	1.27	10	56.3

7.11	1.27	10	57.5
7.11	1.27	10	54.7
7.11	1.27	10	56.0
7.11	3.01	8.5	12.9
7.11	3.01	10	52.7
11.72	0.4	8.5	31.8
11.72	0.4	10	45.4
11.72	1.27	8.5	41.0
11.72	1.27	10	58.8
11.72	3.01	8.5	12.8
11.72	3.01	10	48.2

Capture and concentration

HIC experiment 1

The load was prepared by adding 132 g $(NH_4)_2SO_4$/L refolding mix. A HiTrap Octyl FF column with a volume of 1 mL (GE Healthcare) was used with a flow rate of 150 cm^{-1} h^{-1}. The column was equilibrated with buffer A (20 mM Tris pH 8.5; 7 % v/v glycerol; 1 M $(NH_4)_2SO_4$) and 50 mL load were applied. After the load, a wash step with buffer A (16 CV) was performed. Thereafter, a linear gradient elution was performed with 0-100 % buffer B (20 mM Bis-Tris pH 7; 7 % v/v glycerol) in 30 CV. Volumetric enzyme activity [U/mL] was measured for all fractions.

HIC experiment 2

For HIC experiment 2 the load was prepared by adding 267 g NaCl/L refolding mix. A HiTrap Phenyl FF (High Sub) column with a volume of 1 mL (GE Healthcare) was used with a flow rate of 150 cm^{-1} h^{-1}. The column was equilibrated with buffer A (20 mM Tris pH 8.5; 7 % v/v glycerol; 4 M NaCl) and 50 mL load were applied. After the load, a wash step with buffer A (9 CV) was performed. Thereafter, a linear gradient elution was performed with 0-100 % buffer B (20 mM Bis-Tris pH 7; 7 % v/v glycerol) in 30 CV. Volumetric enzyme activity [U/mL] was measured for all fractions.

HIC experiment 3

For HIC experiment 3 the load was prepared by adding 267 g NaCl / L refolding mix. A HiTrap Phenyl FF (High Sub) column with a volume of 1 mL (GE Healthcare) was used with a flow rate of 75 cm^{-1} h^{-1}. The column was equilibrated with buffer A (20 mM Tris pH 8.5; 7% v/v glycerol; 4 M NaCl) and 50 mL load were applied. After the load, a wash step with buffer A (10 CV) was performed. Thereafter, a step elution was performed with 60 % buffer B (20 mM Bis-Tris pH 7; 7 % v/v glycerol / 9 CV), 90 % buffer B (26 CV) and 100 % (32 CV) buffer B, with active HRP eluting at 90 % buffer B. Volumetric enzyme activity [U/mL] was measured for all fractions.
